# Core outcome set for symptomatic uncomplicated gallstone disease

**DOI:** 10.1093/bjs/znac095

**Published:** 2022-04-05

**Authors:** Karen Innes, Jemma Hudson, Katie Banister, Bernard Croal, Craig Ramsay, Irfan Ahmed, Jane Blazeby, Katie Gillies

**Affiliations:** Health Services Research Unit, University of Aberdeen Institute of Applied Health Sciences, Aberdeen, UK; Health Services Research Unit, University of Aberdeen Institute of Applied Health Sciences, Aberdeen, UK; Health Services Research Unit, University of Aberdeen Institute of Applied Health Sciences, Aberdeen, UK; Clinical Biochemistry, Grampian University Hospitals NHS Trust, Aberdeen, UK; Health Services Research Unit, University of Aberdeen Institute of Applied Health Sciences, Aberdeen, UK; Department of Surgery, NHS Grampian, Aberdeen, UK; Department of Social Medicine, University of Bristol, Bristol, UK; Health Services Research Unit, University of Aberdeen Institute of Applied Health Sciences, Aberdeen, UK

## Abstract

**Background:**

Heterogeneity of outcomes is a problem for assessing intervention effectiveness when considering treatments for uncomplicated symptomatic gallstone disease. The value to all stakeholders of outcomes that have been measured and reported to date is also unclear. The aim of this study was to develop a core outcome set for symptomatic uncomplicated gallstone disease.

**Methods:**

An in person-meeting was held with patients to prioritize potentially important outcomes from a previously developed longlist of outcomes. This was followed by an online three-round Delphi survey that was conducted with healthcare professionals. The results of each consensus process were compared and combined to produce the final core outcome set.

**Results:**

A total of 82 participants enrolled in round 1 of the Delphi survey, with a final sample of 40 participants contributing to round 3. Five patients contributed to the in-person group meeting. Following the consensus processes, 11 outcomes were considered to be core by patients and healthcare professionals, and included in the core outcome set. These were: quality of life; overall health state; overall satisfaction; overall pain; common bile duct injury; biliary leak; haemorrhage; need for endoscopic retrograde cholangiopancreatography; intra-abdominal collections; admission/readmission for problems; and reoperation.

**Conclusion:**

A core outcome set for symptomatic uncomplicated gallstone disease has been developed with patients and healthcare professionals. Eleven outcomes across four key domains have been identified. These represent the minimum set of outcomes that should be reported in trials evaluating interventions for gallstone disease.

## Introduction

Gallstone disease (cholelithiasis) is one of the most common gastrointestinal diseases worldwide, with a prevalence of approximately 10–15 per cent in adult populations, but more common in women and people aged over 40 years^1^. For the majority (around 80 per cent), gallstones are asymptomatic, but 20 per cent of people experience pain and develop gallstone-related complications. These patients are currently offered symptom control and/or surgical intervention. A significant number of these patients (up to 40 per cent) remain symptomatic after intervention^2^. Conducting large, well designed, RCTs comparing surgery with non-surgical comparators, which measure and report outcomes that matter to all stakeholders, is of importance in this setting.

A recent in-depth analysis^3^ of outcomes that had been reported or were considered relevant to symptomatic gallstone disease highlighted the significant variability between studies. In addition, outcome mapping also included empirical research that identified new, unreported in this context, outcomes of importance^3^. This heterogeneity, and absence of outcomes deemed important to patients (and other stakeholders), brings into question the adequacy of existing evidence. Standardizing outcomes through the development and implementation of core outcome sets (COSs) offers a potential solution to this heterogeneity problem and ensures that the right outcomes are being measured and reported^4^. COSs aim to define a minimum set of outcomes that should be considered essential for the evaluation and reporting of specific interventions or conditions^4^.

This study aimed to develop a COS for uncomplicated symptomatic gallstone disease effectiveness trials, which recommends what outcomes should be measured and reported as a minimum, reflecting the interests of relevant stakeholders to facilitate decision-making.

## Methods

The study was designed using existing best-practice approaches for COS development and included three linked phases^4^: the generation of a longlist of outcomes informed by a systematic review of quantitative and qualitative evidence, and qualitative interviews with patients; a face-to-face meeting with patients to determine which outcomes they considered to be core; and a three-round Delphi survey with healthcare professionals to determine the final COS. The study was registered in the COMET Initiative database^5^. The COS was developed alongside an ongoing RCT comparing laparoscopic cholecystectomy with observation/conservative management for preventing recurrent symptoms and complications in adults with uncomplicated symptomatic gallstones (C-Gall trial, ISRCTN55215960). The scope of this COS was restricted to interventions (both surgical and non-surgical) for the treatment of uncomplicated symptomatic gallstone disease in adults.

### Phase 1: generation of outcome list

This phase of outcome mapping has been published elsewhere^3^. In brief, a systematic literature review was conducted to identify outcomes reported in trials of interventions for symptomatic uncomplicated gallstone disease, and outcomes from exploratory studies reported by patients with a lived experience of symptomatic uncomplicated gallstone disease. In addition, a content analysis of individual items in disease-specific patient-reported outcome measures (PROMs) was conducted. Outcomes of relevance to patients were identified from analysis of interviews and focus groups with patients who had a diagnosis of symptomatic uncomplicated gallstone disease, and audio recordings of consultations for a clinical trial evaluating treatments for symptomatic uncomplicated gallstone disease.

### Phase 2: face-to-face meeting with patients

Patients who were members of the C-GALL Patient Involvement Group (established to provide patient input into the C-GALL trial; required patients to have had a diagnosis of gallstone disease and received either surgical or non-surgical management) were invited to contribute to the meeting. A face-to-face meeting with patients only was chosen for a number of reasons: given previous experience of trying to identify patients for the C-GALL Patient Involvement Group, it was known this was not a straightforward task and the process yielded low numbers; it was felt that the existing patient group would feel more able to comment on outcomes without the input of healthcare professionals to influence decisions; and the authors wanted to have the opportunity to understand any misunderstandings about the COS or the outcomes.

In advance of the meeting, patients were e-mailed information, including a brief description of COSs and the purpose of the activity. Patients were also provided with the list of 54 identified outcomes from phase 1, and asked to identify their top three outcomes from the list to discuss at the meeting. The group discussion was facilitated by the Health Services Research Unit (HSRU) Patient Public Involvement (PPI) Coordinator and the C-GALL trial PPI co-applicant. Verbal consent was sought at the start of the meeting, and all discussion was audio-recorded. Each individual shared their top three identified outcomes, with a short explanation of why they felt each was important. All 15 outcomes that were identified by the group as important were then discussed further, allowing each participant to put forward their opinions and perspectives about the outcome. After discussion, the individuals rated the 15 outcomes as being of high, medium or lower importance. It was stressed that these were all likely to be important, but the purpose of the activity was to focus on the *most* important, which should be collected every time. The outcomes were then ranked based on the average scoring and the consensus definition applied (more than 95 per cent scoring high) to determine inclusion of outcomes in the core set. After all 15 identified outcomes had been discussed, the group was encouraged to check whether there were any other outcomes they felt would be important to include. This provided opportunities for further clarification of outcomes and discussion of outcomes that participants felt should not be included in the COS.

### Phase 3: online Delphi survey with healthcare professionals

A Delphi survey was used to seek agreement on the relative importance of outcomes identified in phases 1 and 2. Each outcome generated in phase 1 was listed together with a plain-language definition on the online DelphiManager platform^6^. Although there is no formal guidance on sample size for the panel in Delphi surveys, a minimum of 10–18 has been suggested, and, as such, the aim was to have a final sample larger than 18^7,8^. Healthcare professionals were invited to participate via e-mail distribution lists of professional societies and through social media. Specifically, the Association of Surgeons of Great Britain, Association of Upper Gastrointestinal Surgery of Great Britain and Ireland, and Association of Laparoscopic Surgeons of Great Britain and Ireland were asked to send the e-mail invitation to their memberships. General practitioners at Scottish sites involved in the C-GALL trial were sent invitations through the Primary Care Network.

An e-mail invitation contained a brief information sheet and a link to the Delphi website, providing further information and allowing interested participants to register. Two rounds of the survey (R1 and R2) were completed. In R1, the outcomes were listed alphabetically by domain, and participants asked to score their importance using a nine-point Likert scale, where 1–3 indicated not important, and 7–9 essential. Participants were also invited to submit any additional outcomes during R1, which were reviewed by the study team and considered for inclusion in R2. All participants were asked to complete R2. Participants were provided with their R1 score and an anonymized distribution of the group’s scores. Participants were asked to consider this information when scoring the outcome again in R2. A final round of rating (R3) was completed, with feedback provided as in R2. However, R3 also provided responders with patients’ scores from phase 2, and asked them to consider the importance of that outcome in light of their own scores, the scores of other clinicians, and the scores of patients. No outcomes were removed between the rounds.

### Consensus definition

The original consensus definition was based on existing COS studies, which required 70 per cent or more of the group to agree an outcome as important (or not), and less than 15 per cent score in the opposite direction^9,10^. However, while blinded to outcome identity following R3, the consensus definition was amended given the large number of outcomes meeting the original consensus definition and considered ‘consensus in’ (i.e. 21). The stringency for ‘consensus in’ outcomes being included in the core set was increased to more than 95 per cent scoring 7–9, and 50 per cent or less scoring 7–9 as ‘consensus out’ across the Delphi survey, and more than 95 per cent scoring high importance in the patient consensus meeting.

Following the Delphi process, the outcomes that had reached consensus from the patient meeting were compared and combined with the outcomes meeting consensus for inclusion in the core set from the Delphi survey.

### Research ethics

This study was approved as part of the C-GALL trial by the North of Scotland Research Ethics Service (16/NS/0053) and National Health Service (NHS) Grampian Research and Development. Verbal consent was sought from face-to-face meeting participants, and consent was implicit by completion and return of the Delphi survey.

### Patient and public involvement

The HSRU PPI Partnership contributed to the development of a lay description for clinical outcomes—a section explaining in lay terms the clinical description and, in addition, a section ‘what this means for patients’ to explain the rationale for the outcome.

## Results

**Fig. 1:**
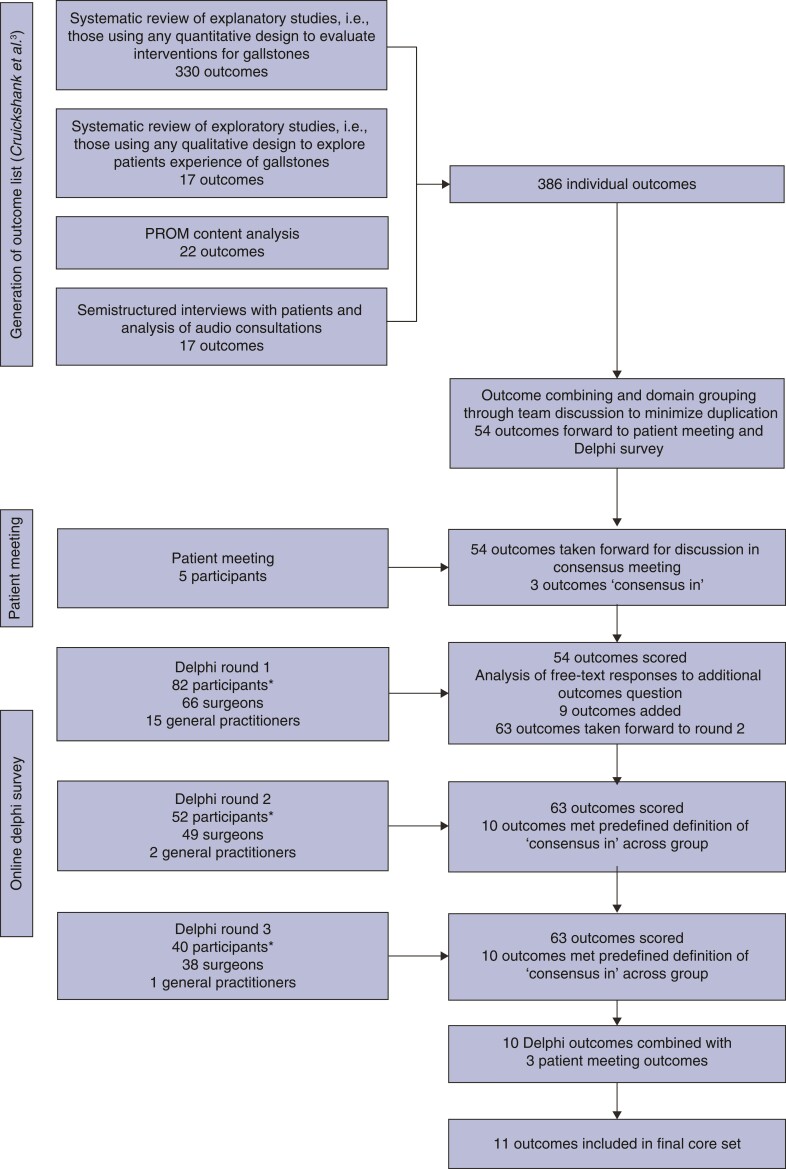
Core outcome set development overview *One Delphi participant did not specify a clinical role. PROM, patient-reported outcome measure.

Phase 1 has been published in detail elsewhere^3^, so this paper focuses on the reporting of phases 2 and 3. A total of 54 outcomes, with lay descriptions, were presented to participants in the face-to-face meeting and Delphi survey (*Fig. 1* and *Table S1*).

### Phase 2: face-to-face meeting with patients

Five patients with uncomplicated gallstone disease, three of whom had previously undergone surgery (2 with resolution of symptoms, one without) and two patients who had not had surgery, participated in the meeting. After sharing of prioritized outcomes, the group agreed that three outcomes should be considered as having high importance for inclusion in a COS: quality of life, overall pain, and overall health state. When discussing overall health state, there were also discussions about anxiety. The group felt that any measurement of overall health state would be more important than anxiety alone, but that it was important to take into consideration the anxiety aspects of the disease when measuring overall health state, and overall mental strain when dealing with the symptoms and length of time waiting for diagnosis and treatment (*Table S2*).

### Phase 3: online Delphi survey with healthcare professionals

#### Sample characteristics

Round 1 of the Delphi survey was completed by 82 participants (66 surgeons and 15 general practitioners and one participant who did not specify a clinical role) and R2 by 54 (65 per cent of those from R1). The R3 participant sample comprised 38 surgeons, one general practitioner and one respondent who did not specify a clinical role (a total of 70 per cent of those from R2) (*Table 1*). The majority were men, aged 45–64 years, and based in England. The Delphi survey was open to responses across the three rounds from June 2019 to January 2021.

**Table 1:** Delphi sample demographics (Round 3)

	No of participants (*n* = 40)
**Clinical role**	
Surgeon	38
General practitioner	1
Unknown	1
**Sex**	
M	33
F	7
**Age (years)**	
18–44	17
45–64	22
65–84	1
**Place of residence**	
England	34
Scotland	4
Wales	2
**Experience in treating uncomplicated gallstones**	
1–3 years	33
Missing	7

After R1 scoring, 41 additional outcomes were suggested for consideration. Following discussion with the study team, nine were agreed as new and taken forward for scoring in R2 and R3 (these were not scored by patients during the meeting). These additional nine outcomes included: readmission; reoperation; need for endoscopic retrograde cholangiopancreatography; percutaneous drain; use of analgesia; need for an outpatient appointment; steatorrhoea; pancreatitis; and appetite. The other suggested outcomes were excluded because they were out of scope or duplicates of existing outcomes.

Scores from 40 participants in R3 were included in the final analysis, an attrition rate of 37 per cent from R1, and 23 per cent from R2. Attrition analysis indicated no significant difference between responders and non-responders between the Delphi rounds.

#### Core outcome set

At the end of R3 of the Delphi process, 10 outcomes achieved consensus for inclusion in the COS. Two of these had also been identified as ‘consensus in’ from the patient meeting. An additional outcome (overall pain) was also identified as consensus in during the patient meeting but did not achieve consensus in during the healthcare professional Delphi. The final COS includes 11 outcomes grouped across four domains (*Table 2*). These are: quality of life; overall health state; overall satisfaction; overall pain; common bile duct injury; biliary leak; haemorrhage; need for endoscopic retrograde cholangiopancreatography; intra-abdominal collections; admission/readmission for problems; and reoperation. It is important to note that, owing to the inclusion of both surgical and non-surgical interventions in the scope of the COS, some of the outcomes in this set are relevant only to surgical interventions; as such, these particular outcomes (marked ‡ in *Table 2*) would not require reporting in trials of non-surgical interventions only using this COS.

**Table 2:** Core outcome set for uncomplicated symptomatic gallstone disease

Outcome	Domain
Quality of life*	Generic health
Overall health state*	
**Overall satisfaction**	
**Overall pain**†	
**Common bile duct injury**‡	Intraoperative adverse event
**Biliary leak**‡	
**Haemorrhage**‡	
**Need for ERCP**‡	
**Intra-abdominal collections**‡	Intraoperative and postoperative adverse event
**Admission/readmission for problems**	Cost-effectiveness
**Reoperation**	

ERCP, endoscopic retrograde cholangiopancreatography. *Identified as core by patient meeting and healthcare professional Delphi survey. †Identified as core by patient meeting only. ‡Outcome relevant to surgical interventions only.

## Discussion

In recent years, there has been a significant increase in the development of COSs for use in effectiveness trials comparing surgical approaches with a range of comparators. For example, the COMET registry^11^ contains 183 entries including the word ‘surgery’ in its database, covering a broad range of specialties from cancer to trauma and cosmetic surgery. The value of the COS developed in the project reported here is for trials of interventions to treat uncomplicated gallstone disease, for which there have been many studies evaluating various interventions^12^. It is also important to reiterate that the development of this COS does not preclude the measurement and reporting of other outcomes when evaluating interventions for the treatment of uncomplicated gallstone disease. However, the inclusion of additional outcomes should be considered with regard to resource and burden for those tasked with collecting and reporting. Although the COS reported here was designed for use in effectiveness trials, like many other COSs, both in surgery and other specialties, it could have value in research and clinical practice^13,14^.

COSs focus on the ‘what’ of outcome measurement and reporting, rather than the ‘how’, which would include defining time points at which the measurement should be made. Therefore, future research should determine how to measure the outcomes identified in this COS. Some of this work has already been conducted. A recent systematic review^15^ assessing the methodological quality of PROMs in patients undergoing laparoscopic cholecystectomy identified six validated measures. The review was not able to recommend a specific PROM for use in laparoscopic cholecystectomy because of the limited number of studies and poor quality of the measures identified^15^. A more recent review^16^ also identified considerable variation in the measurement and reporting of patient-reported outcomes after laparoscopic cholecystectomy. This more recent review identified the need for a COS that would incorporate patient-reported outcomes and the consideration of longer-term outcomes^16^.

It is important to highlight that one of the outcomes (overall pain) included in the COS was identified in the primary qualitative research and is the only outcome to be brought into the COS by the rating of the patient group only. The other two outcomes scored as important by the patient group were also agreed as ‘consensus in’ during the healthcare professional Delphi survey. This demonstrates the value of including patients during the COS development process.

The final COS included several surgical complications. Typically, surgeons are very focused on complications as an outcome, even though these are classified as adverse events in pharmaceutical trials. Adverse events are inevitable with any healthcare intervention and need to be considered alongside treatment benefits. In evaluating surgery, it is difficult to balance the procedure-specific adverse events with more generic complications (such as wound/chest infection). All may have impacts on patients’ long-term quality of life and health. Other COSs have faced similar challenges with categories of outcomes after surgical procedures^17^. In the present study, because these endpoints were prioritized by stakeholders, they were retained in the COS. It is noted that national audits highlight the frequency of these problems, and also underline their importance. Future work will need to examine how the adverse events experienced in the short term are associated with long-term clinical benefits.

This study does have some weaknesses. Given the challenges with recruitment and sustained participation in the Delphi process, it will be important to engage with a wide range of healthcare professionals and trialists to ensure that this COS for uncomplicated symptomatic gallstone disease is implemented as widely as possible. Further work may also be required among additional clinical stakeholder groups, such as general practitioners and gastroenterologists, to ensure that the core outcomes represent outcomes they would also consider as core when evaluating a range of interventions for the treatment of uncomplicated symptomatic gallstones. In addition, determining the transferability of this COS to other settings, such as low- and middle-income countries, is also important to ensure wide applicability and implementation. In addition, the small number of patients included in the rating of outcomes, and a lack of opportunity to share the opinions of healthcare professionals with patients, is also a limitation.

A key strength of the work is the extensive outcome mapping exercise on which the COS builds. Even though a small number of patients contributed to the face-to-face meeting, outcomes identified as of importance to participants in the list of outcomes for scoring in the Delphi was informed by an extensive synthesis of patient reported relevant outcomes through systematic literature review and primary research^3^.

The study has developed the first COS for use in effectiveness trials evaluating interventions for uncomplicated symptomatic gallstone disease. It was registered prospectively on the COMET Initiative database, and development and reporting have been informed by existing standards for COSs^4,5^. The final COS includes 11 outcomes deemed critically important by both patients and healthcare professionals.

## Supplementary Material

znac095_Supplementary_Data

## References

[1] Barbara L, Sama C, Morselli Labate AM, Taroni F, Rusticali AG, Festi D et al A population study on the prevalence of gallstone disease: the Sirmione Study. Hepatology 1987;7:913–917 3653855 10.1002/hep.1840070520

[2] Festi D, Reggiani ML, Attili AF, Loria P, Pazzi P, Scaioli E et al Natural history of gallstone disease: expectant management or active treatment? Results from a population-based cohort study. J Gastroenterol Hepatol 2010;25:719–724 20492328 10.1111/j.1440-1746.2009.06146.x

[3] Cruickshank M, Newlands R, Blazeby J, Ahmed I, Bekheit M, Brazzelli M et al Identification and categorisation of relevant outcomes for symptomatic uncomplicated gallstone disease: in-depth analysis to inform the development of a core outcome set. BMJ Open 2021;11:e045568 10.1136/bmjopen-2020-045568 PMC8231013 34168025

[4] Williamson PR, Altman DG, Blazeby JM, Clarke M, Devane D, Gargon E et al Developing core outcome sets for clinical trials: issues to consider. Trials 2012;13:132 22867278 10.1186/1745-6215-13-132 PMC3472231

[5] COMET Initiative . https://www.comet-initiative.org/Studies/Details/1909 (accessed 17 March 2022)

[6] COMET Initiative . DelphiManager. http://www.comet-initiative.org/delphimanager/ (accessed 17 March 2022)

[7] Okoli C, Pawlowski SD. The Delphi method as a research tool: an example, design considerations and applications. Inform Manag 2004;42:15–29

[8] Murphy MK, Black NA, Lamping DL, McKee CM, Sanderson CF, Askham J et al Consensus development methods, and their use in clinical guideline development. Health Technol Assess 1998;2:i–iv,1–88 9561895

[9] Harman NL, Bruce IA, Callery P, Tierney S, Sharif MO, O’Brien K et al MOMENT—Management of Otitis Media with Effusion in Cleft Palate: protocol for a systematic review of the literature and identification of a core outcome set using a Delphi survey. Trials 2013;14:70 23497540 10.1186/1745-6215-14-70 PMC3716725

[10] Macefield R, Blencowe N, Brookes S, Jacobs M, Sprangers M, Williamson P et al Core outcome set development: the effect of Delphi panel composition and feedback on prioritisation of outcomes. Trials 2013;14:P77

[11] https://www.comet-initiative.org/Studies/SearchResults (accessed 12 November 2021)

[12] Brazzelli M, Cruickshank M, Kilonzo M, Ahmed I, Stewart F, McNamee P et al Systematic review of the clinical and cost effectiveness of cholecystectomy *versus* observation/conservative management for uncomplicated symptomatic gallstones or cholecystitis. Surg Endosc 2015;29:637–647 25119541 10.1007/s00464-014-3712-6

[13] Remus A, Smith V, Gutke A, Mena JJS, Mørkved S, Wikmar LN et al A core outcome set for research and clinical practice in women with pelvic girdle pain: PGP-COS. PLoS One 2021;16:e0247466 33630941 10.1371/journal.pone.0247466 PMC7906405

[14] Chiarotto A, Ostelo RW, Turk DC, Buchbinder R, Boers M. Core outcome sets for research and clinical practice. Braz J Phys Ther 2017;21:77–84 28460714 10.1016/j.bjpt.2017.03.001 PMC5537457

[15] Daliya P, Gemmill EH, Lobo DN, Parsons SL. A systematic review of patient reported outcome measures (PROMs) and quality of life reporting in patients undergoing laparoscopic cholecystectomy. Hepatobiliary Surg Nutr 2019;8:228–245 31245403 10.21037/hbsn.2019.03.16 PMC6561890

[16] Alexander HC, Nguyen CH, Moore MR, Bartlett AS, Hannam JA, Poole GH et al Measurement of patient-reported outcomes after laparoscopic cholecystectomy: a systematic review. Surg Endosc 2019;33:2061–2071 30937619 10.1007/s00464-019-06745-7

[17] Alkhaffaf B, Metryka A, Blazeby JM, Glenny AM, Adeyeye A Costa PM et al Core outcome set for surgical trials in gastric cancer (GASTROS study): international patient and healthcare professional consensus. Br J Surg 2021;108:1216–1224 10.1093/bjs/znab192 PMC10364901 34165555

