# Supplementary material for: Core outcome set for symptomatic uncomplicated gallstone disease
Source: Br J Surg. 2022 Apr 5;109(6):539–44. doi: 10.1093/bjs/znac095 (PMC10364745; doi:10.1093/bjs/znac095)
Supplement: znac095_Supplementary_Data [file znac095_supplementary_data.zip › Supplementary_Tables.docx]

**Table S1**

| **How important is it that an intervention to treat uncomplicated symptomatic gallstone disease** **can be shown to affect:** | | | |
| --- | --- | --- | --- |
| **#** | **Domain** | **Outcome** | **Definition** |
| **1** | *Physical* | physical activity | Activities such as walking, running, swimming, cycling, physical labour, climbing stairs, gardening etc |
| **2** |  | exercise | Being able to do activities requiring physical effort, carried out to sustain or improve health and fitness (strength and endurance) |
| **3** | *Role* | no of days sick leave | Length of time off work after the operation in days |
| **4** |  | time to everyday life | Length of time taken to return to usual everyday activities |
| **5** |  | impact on others | Impact of your gallstone condition or your gallstone surgery on relationships with people surrounding you |
| **6** | *Pain* | overall pain | Overall pain |
| **7** |  | abdominal pain | General pain occurring at rest and/or when coughing, originating in the abdominal area |
| **8** |  | umbilical pain | Pain around the belly button scar (this is where the main port is that removed the stones) |
| **9** |  | shoulder pain | Pain relating to or affecting the right shoulder region |
| **10** | *Bowel movements* | diarrhoea | Watery stools, loose bowel motion |
| **11** |  | constipation | Difficulty passing stool |
| **12** | *Thirst/dehydration* | resumption of orals | Starting to eat and drink after treatment |
| **13** | *Appetite/eating/taste* | time to resume eating | Length of time taken to return to oral food intake |
| **14** | *Fatigue* | fatigue | Feeling physically or mentally tired or lacking in energy |
| **15** | *Sleep* | length of night sleep | Length of night’s sleep |
| **16** | *Cognitive* | difficulty concentrating | Inability to focus attention on one task or problem |
| **17** | *Emotional* | anxiety | A feeling of worry, nervousness or unease |
| **18** |  | distress | A feeling of extreme anxiety, stress or anguish |
| **19** |  | trust | belief in the reliability, truth, or ability of someone or something |
| **20** | *Generic health* | quality of life | How well you feel physically and emotionally because of a combination of:   - Your gallstones - The prospect of treatment - The result of treatment (Treatment might include surgery or painkillers) |
| **21** |  | overall health state | Overall state of your physical and mental condition |
| **22** |  | overall satisfaction | The degree to which expectations or needs have been fulfilled |
| **23** | *Dietary habits* | food intolerance | An adverse physical reaction by the body to certain foods |
| **24** | *Social* | time away from recreational activities | Time spent away from enjoyable activities as a result of your gallstone condition or gallstone surgery |
| **25** | *Belching/bloating/gas* | flatulence | Belching, farting, bloating or gas |
| **26** |  | bloating | Abdominal swelling as a result of excess fluid or gas |
| **27** |  | abdominal discomfort | Pain or discomfort in the stomach area |
| **28** | *Service use* | hospital stay | Length of time spent in the hospital from admission to discharge |
| **29** | *Vomiting/nausea* | vomiting | Being sick |
| **30** |  | nausea | Feeling sick |
| **31** | *Reflux* | heartburn | A form of indigestion that presents as a burning sensation in the chest, caused by acid reflux |
| **32** | *Body image* | satisfaction with body image | A feeling of satisfaction with your physical appearance |
| **33** |  | satisfaction with the cosmetic outcome | The extent to which you are content with the cosmetic results of gallstone surgery |
| **34** | *Sexual function* | satisfaction in the context of sexual intercourse | The extent to which you are satisfied with experiences of sexual intercourse in relation to your gallstone condition or your gallstone surgery  *Note: This outcome is particularly relevant to women having Natural Orifice Transluminal Endoscopic Surgery (NOTES)* |
| **35** |  | pain in the context of sexual intercourse | The extent to which you are experiencing pain during or after sexual intercourse in relation to your gallstone condition or your gallstone surgery  *Note: This outcome is particularly relevant to women having Natural Orifice Transluminal Endoscopic Surgery (NOTES)* |
| **36** | *Regurgitation* | regurgitation | Bringing swallowed food back up to the mouth |
| **37** | *Dysphagia/swallowing* | trouble swallowing food | Problems swallowing food |
| **38** | *Generic symptoms* | general discomfort | An unpleasant feeling and/or low-level pain which is hard to define |
| **39** |  | residual symptoms | Continuing to have symptoms (such as pain, bloating, etc.) after removal of the gallbladder |
| **40** |  | dizziness | Feeling light-headed or dizzy |
| **41** |  | fainting | Fainting (short-term loss of consciousness) |
| **42** | *Mortality* | mortality | Death from any cause |
| **43** | *Intra-op ae* | Common bile duct stones | Stones in the common bile duct |
| **44** |  | Common bile duct injury | During surgery the common bile duct is damaged |
| **45** |  | biliary leak | The liver produces bile which is stored in the gallbladder (see diagram). If this is damaged, the bile can leak and cause complications. |
| **46** |  | haemorrhage | Bleeding or the abnormal flow of blood; the release of blood from a ruptured blood vessel |
| **47** | *Intra and post-op ae* | intra-abdominal collections | After surgery, any type of fluid collecting in the abdomen. |
| **48** | *Post-op ae* | hernia occurrence | internal hernia – displacement of an organ within the abdomen through a potential defect. |
| **49** |  | port-site complications | Complications such as infection, hernia, pain or bleeding at or within the ‘keyholes’ characteristic of keyhole surgery |
| **50** |  | wound infections | An infection at the wound site |
| **51** |  | patient-perceived success of the operation | How patients perceive the success of the operation |
| **52** | *Cost-effectiveness* | hospital cost | Total hospital costs, taking into account the total length of hospital stay, operating room charges, medical and surgical supplies, pharmacy, laboratory and pathology, recovery room, anaesthesia, and ICU/observation rooms |
| **53** |  | overall cost | Cost of use of healthcare services; e.g. contact with a GP, in- or outpatient contact, prescribed medications |
| **54** |  | cost-effectiveness ratio | Cost-effectiveness of treatment route (medical management or surgery to remove the gallbladder), calculated by dividing cost by success rate  (defined by the quality of life after treatment) |

**Table S2**

| **Outcome (No.)** | **Ranking (individual)** | **Ranking overall** | **Order discussed** |
| --- | --- | --- | --- |
| Quality of life (20) | HHHHH | HIGH | 1 |
| Overall Pain (6) | HHHHH | HIGH | 6 |
| Overall Health State*(21) | HHHHH | HIGH | 11 |
| Physical Activity (1) | HHHHM | MEDIUM | 4 |
| Fatigue (14) | HHHMM | MEDIUM | 10 |
| Diarrhoea (10) | HHMMM | MEDIUM | 9 |
| Anxiety (17) | HHMMM | MEDIUM | 12 |
| Food intolerance (23) | HMMMM | MEDIUM | 2 |
| Nausea (40) | M/H MMMM | MEDIUM | 15 |
| Flatulence (25) | MMMLL | LOWER | 7 |
| CBD Injury (54) | H M/H LLL | LOWER | 5 |
| Constipation (11) | MMLLL | LOWER | 8 |
| Bloating (36) | MLLLL | LOWER | 3 |
| Mortality (52) | Not felt it should be included – low risk perceived |  | 13 |
| Residual symptoms (49) | Not felt it should be included – already covered in other |  | 14 |

H=high,m=medium, l=lower (M/H medium to high)
