# Supplementary material for: Core outcome set for symptomatic uncomplicated gallstone disease
Source: Br J Surg. 2022 Apr 5;109(6):539–44. doi: 10.1093/bjs/znac095 (PMC10364745; doi:10.1093/bjs/znac095)
Supplement: znac095_Supplementary_Data [file znac095_supplementary_data.zip › COS-STAR_Checklist.docx]

**Core Outcome Set-STandards for Reporting: The COS-STAR Statement Checklist**

*A core outcome set for symptomatic uncomplicated gallstone disease.*

| **SECTION/TOPIC** | **ITEM No.** | **CHECKLIST ITEM** | **REPORTED ON PAGE NUMBER** |
| --- | --- | --- | --- |
| TITLE/ABSTRACT | | | |
| Title | 1a | Identify in the title that the paper reports the development of a COS | 1 |
| Abstract | 1b | Provide a structured summary | 2 |
| INTRODUCTION | | | |
| Background and Objectives | 2a | Describe the background and explain the rationale for developing the COS. | 3 |
|  | 2b | Describe the specific objectives with reference to developing a COS. | 3 |
| Scope | 3a | Describe the health condition(s) and population(s) covered by the COS. | 3 |
|  | 3b | Describe the intervention(s) covered by the COS. | 3 |
|  | 3c | Describe the setting(s) in which the COS is to be applied. | 3 |
| METHODS | | | |
| Protocol/Registry Entry | 4 | Indicate where the COS development protocol can be accessed, if available, and/or the study registration details. | 3 |
| Participants | 5 | Describe the rationale for stakeholder groups involved in the COS development process, eligibility criteria for participants from each group, and a description of how the individuals involved were identified. | 4 |
| Information Sources | 6a | Describe the information sources used to identify an initial list of outcomes. | 3 and ref 3 |
|  | 6b | Describe how outcomes were dropped/combined, with reasons (if applicable). | 3 and ref 3 |
| Consensus Process | 7 | Describe how the consensus process was undertaken. | 4 and 5 |
| Outcome Scoring | 8 | Describe how outcomes were scored and how scores were summarised. | 4 and 5 |
| Consensus Definition | 9a | Describe the consensus definition. | 5 |
|  | 9b | Describe the procedure for determining how outcomes were included or excluded from consideration during the consensus process. | 4 and 5 |
| Ethics and Consent | 10 | Provide a statement regarding the ethics and consent issues for the study. | 5 |
| RESULTS | | | |
| Protocol Deviations | 11 | Describe any changes from the protocol (if applicable), with reasons, and describe what impact these changes have on the results. | 5 |
| Participants | 12 | Present data on the number and relevant characteristics of the people involved at all stages of COS development. | Table 1 and page 6 |
| Outcomes | 13a | List all outcomes considered at the start of the consensus process. | Supplementary Table 1 |
|  | 13b | Describe any new outcomes introduced and any outcomes dropped, with reasons, during the consensus process. | 6 |
| COS | 14 | List the outcomes in the final COS. | 6 and 7 |
| DISCUSSION | | | |
| Limitations | 15 | Discuss any limitations in the COS development process. | 7 |
| Conclusions | 16 | Provide an interpretation of the final COS in the context of other evidence, and implications for future research. | 7 |
| OTHER INFORMATION | | | |
| Funding | 17 | Describe sources of funding/role of funders. | 1 |
| Conflicts of Interest | 18 | Describe any conflicts of interest within the study team and how these were managed. | 1 |

*From: Kirkham JJ, Gorst S, Altman DG, Blazeby JM, Clarke M, Devane D, et al. (2016) Core Outcome Set–STAndards for Reporting: The COS-STAR Statement. PLoS Med 13(10): e1002148. https://doi.org/10.1371/journal.pmed.1002148*
